# CTA095, a Novel Etk and Src Dual Inhibitor, Induces Apoptosis in Prostate Cancer Cells and Overcomes Resistance to Src Inhibitors

**DOI:** 10.1371/journal.pone.0070910

**Published:** 2013-08-15

**Authors:** Wenchang Guo, Ruiwu Liu, Gaurav Bhardwaj, Ai-Hong Ma, Chun Changou, Joy C. Yang, Yuanpei Li, Caihong Feng, Yan Luo, Anisha Mazloom, Eduardo Sanchez, Yan Wang, Wenzhe Huang, Randen Patterson, Christopher P. Evans, Kit S. Lam, Hsing-Jien Kung

**Affiliations:** 1 Department of Biochemistry and Molecular Medicine, University of California Davis, Sacramento, California, United States of America; 2 Department of Physiology and Membrane Biology, University of California Davis, Sacramento, California, United States of America; 3 Department of Urology, University of California Davis, Sacramento, California, United States of America; 4 National Health Research Institutes (NHRI), Miaoli County, Taiwan, ROC; University of Pittsburgh School of Medicine, United States of America

## Abstract

Etk is a non-receptor tyrosine kinase, which provides a strong survival signal in human prostate cancer cells. Src, another tyrosine kinase that cross-activates with Etk, has been shown to play an important role in prostate cancer metastasis. Herein, we discovered a new class of Etk inhibitors. Within those inhibitors, CTA095 was identified as a potent Etk and Src dual inhibitor. CTA095 was found to induce autophagy as well as apoptosis in human prostate cancer cells. In addition, CTA095 inhibited HUVEC cell tube formation and “wound healing” of human prostate cancer cells, implying its role in inhibition of angiogenesis and metastasis of human prostate cancer. More interestingly, CTA095 could overcome Src inhibitor resistance in prostate cancer cells. It induces apoptosis in Src inhibitor resistant prostate cancer cells, likely through a mechanism of down regulation of Myc and BCL2. This finding indicates that simultaneously targeting Etk and Src could be a promising approach to overcome drug resistance in prostate cancer.

## Introduction

Prostate cancer is the most frequently diagnosed cancer and the second leading cause of cancer deaths of men in the U.S. [1]. While early phase prostate cancer (CaP) can effectively be controlled by hormone therapy, metastatic CaP remains incurable. Tyrosine kinase inhibitors (TKIs) are among the most promising targeted therapies; yet their potential as prostate cancer therapeutics have not been fully realized and, to date, the outcomes of clinical trials using TKIs as single agents have generally been modest, probably due to redundancy in receptor binding and signaling to intracellular mediators [2]. Most of the TKIs that have been developed are directed against receptor tyrosine kinases. Etk is a non-receptor tyrosine kinase, which is over-expressed in human prostate cancer specimens and provides strong survival functions in prostate cancer cells [3], [4]. Etk mediates critical activation of STAT3 in CaP suggesting that functional disruption of Etk may attenuate multiple key signals involved in CaP growth and survival [5]. Etk also regulates survival [6], metastasis [7], drug resistance [3], [8], angiogenesis [9], and apoptosis [10]. Overexpression of Etk induces prostate intraepithelial neoplasia in a mouse [11]. Recent reports indicate that Etk plays an important role in the self-renewal and tumorigenic potential of glioblastoma stem cells through Stat3 activation [12]. Therefore, systemic inhibition of Etk may offer synergistic anti-tumor effects. As of yet, there is no efficacious inhibitor of this kinase.

Src, Etk, and FAK associate with and cross-activate each other. Inhibition of one often decreases the activity of the others. These three kinases have been shown to play an important role in angiogenesis and metastasis of prostate cancer cells. The Src inhibitor, AZD0530, has been reported to inhibit prostate cancer bone metastasis in animal models. However, this inhibitor lacks the activity to induce apoptosis of prostate cancer cells. Dual inhibition of Etk and Src could not only overcome the disadvantage of Src inhibitors, but may also increase efficacy in inhibiting metastasis of prostate cancer cells.

Autophagy is a catabolic process involving the degradation of a cell's own components through the lysosomal machinery [13]. It is a tightly regulated process that helps maintain a balance between the synthesis, degradation, and subsequent recycling of cellular products [14]. Autophagy could contribute to both cell survival and cell killing in a context dependent manner. Autophagy modulators have now emerged as important sensitizers or modifiers of targeted therapy [15], [16].

Herein, we report identification of a novel Etk and Src dual inhibitor, CTA095, which induces autophagy and apoptosis, as well as synergistic effects with autophagy modulators in prostate cancer cells. To our knowledge, this is the first report of an Etk and Src dual inhibitor with an application as an anti-cancer agent.

## Materials and Methods

### Reagents

Purified Etk, Btk, Mertk, Yes and Src kinases were obtained from Millipore Inc (Dundee, UK). Propidium Iodide (PI), *N*,*N*-diisopropylethylamine (DIEA), *N*,*N*-dimethylformimide (DMF), ethanol, acetonitrile (ACN), 1-(3-aminopropyl)piperidine, trifluoroacetic acid (TFA), Pd/C, ammonium formate and dimethyl sulfoxide (DMSO) were purchased from Sigma-Aldrich (Saint Louis, MO). L-Phenylglycine methyl ester hydrochloride was purchased from Chem-Impex International Inc (Wood Dale, IL). Dibenzofuran-2-carboxaldehyde was purchased from Oakwood Products, Inc (West Columbia, SC). The annexin V-FITC apoptosis detection kit was obtained from Abcam (Cambridge, MA). Reversed-phase high-performance liquid chromatography (RP-HPLC) from the Waters Corporation (Milford, MA) was used for analysis and purification of CTA095. LNCAP, CWR22Rv1, RWPE1, 293 and HUVEC cells were obtained from ATCC (Manassas, VA).

### One-pot synthesis of CTA095

The synthetic approach of CTA095, 2-(dibenzo[*b*,*d*]furan-2-yl)-7-phenyl-1-(3-(piperidin-1-yl)propyl)-1*H*-imidazo[4,5-*g*]quinoxalin-6(5*H*)-one, is similar to the approach we previously reported [17]. In brief, a solution of L-Phenylglycine methyl ester hydrochloride (201.7 mg, 1.0 mmol) and DIEA (383.2 μL, 2.2 mmol) in DMF (1.5 ml) was added dropwise under vigorous stirring to a solution of 1,5-difluoro-2,4-dinitrobenzene (204.0 mg, 1.0 mmol) in DMF (0.5 ml). The reaction solution was stirred at room temperature for 45 min. This was followed by the addition of a solution of 1-(3-aminopropyl) piperidine (159 μL, 1.0 mmol) and DIEA (174.2 μL, 1.0 mmol) in DMF (1 ml). The resulting mixture was agitated at room temperature overnight. Ethanol (20 ml), Pd/C (10%, 200 mg), and ammonium formate (1.50 g, 23.8 mmol) were added to the solution. The solution was heated to reflux for 3 h and then cooled to room temperature. The Pd/C was filtered out and the filtrate was concentrated with rotary evaporator. Dibenzofuran-2-carboxaldehyde (196.2 mg, 1.0 mmol) in DMF was added to the solution. The resulting solution was stirred at room temperature for 1 day. The DMF solution was poured into 40 mL of ice. The precipitate was collected by filtration and washed with water, followed by RP-HPLC purification. The fraction was collected and lyophilized to give a yellow powder as the final product (Figure S1). The homogeneity of the compound was checked by analytical RP-HPLC. The purity was determined to be >95% pure. The identity of the compounds was confirmed by matrix-assisted laser desorption/ionization-time of flight mass spectrometry. Found: 554.26 Dalton (calculated: 554.26 Dalton for MH^+^).

### Molecular modeling

Molecular docking studies were performed to understand the binding of CTA095 to Etk. Energy optimized structure of CTA095 was calculated using Merck Molecular Force Field (MMFF) [18], as implemented in Marvin Suite v 5.11 (http://www.chemaxon.com/products/marvin). The three-dimensional structure of human Etk (residues 275–675) sequence (NCBI GI: 42544182) was predicted using a homology modeling based approach as implemented in HHPred [19], [20]. To define a putative binding site for CTA095 on the Etk protein structure, we first used a blind docking approach implemented on the SwissDock webservice (http://swissdock.vital-it.ch) [21], [22]. Among the clusters generated by SwissDock, the conformation with the lowest binding free energy was selected. In order to further understand the stability and dynamics of the ligand-kinase complex, we performed a 20 ns molecular dynamics (MD) simulation, starting with the best-docked structure predicted by SwissDock. These simulations were performed using NAMD v 2.7b2 [23]. CHARMM27 force fields were used to calculate the potentials of Etk, while CHARMM22 (as implemented in SwissParam (http://swissparam.ch) [24] force fields were used to calculate the potentials of CTA095. The kinase-ligand complex was solvated in a water box with periodic boundary conditions. Dimensions of the water box were selected to be at least 10 angstroms larger than the solute in every direction. The whole system was neutralized with 0.15 M NaCl. An initial minimization step was performed for 6000 steps, followed by 20 ns of relaxation. Trajectories of these simulations were visualized using VMD v 1.9 [25], and the interactions between kinase and the ligand were plotted using PyMol and LigPlot+ v 1.4 [26].

### Kinase inhibition assay

Kinase inhibition was measured using thin-layer-chromatography (TLC). Briefly, purified kinases (20 nM), the corresponding substrate (500 µM, TSFYGRH for Etk, YIYGSFK for the other kinases), and CTA095 (0–10 µM) were incubated in a kinase reaction (100 mM Hepes, pH 7.4, 10 mM MnCl_2_, 10 mM MgCl_2_, 1 mM DTT) for 5 min, and the reaction was started by adding 5 μCi ^33^P-labeled ATP. The reaction (10 µl) was incubated at room temperature for 1 h and was stopped by adding 10 μL H_3_PO_4_. The radioactivity of the peptide substrate was analyzed using TLC as previously described [27].

### Etk autophosphorylation assay

Etk autophosphorylation activity was measured by an in vitro kinase assay. Briefly, purified Etk (100 ng) was mixed with CTA095 in the kinase assay buffer (20 mM Hepes pH 7.55, 10 mM MgCl_2_, 10 mM MnCl_2_, 1 mM DTT, 500 μM Na_3_VO_4_). The cold ATP (5 µM) and hot r-^33^P-ATP (5 µCi) were added to the mixture and the kinase reaction was performed at 30°C for 30 min. The reactions were terminated with a 4X SDS-PAGE sample buffer, and then loaded onto an 8% SDS-polyacrylamide gel for electrophoresis. The gel was vacuum dried and the ETK auto kinase activity was analyzed with a phosphoimager (Biorad).

### Cell culture

LNCAP, PC3, CWR22Rv1, and RWPE1 cells were maintained in RPMI 1640 medium containing 10% fetal bovine serum and 1% penicillin/streptomycin/glutamine.

### Western blotting

Western blotting was performed as described previously [28]. Proteins were detected using the following antibodies: β-actin (Sigma-Aldrich, Saint Louis, MO), Etk (Santa Cruz Inc., Santa Cruz, CA), pEtk; Src, pSrc; Stat3, pStat3. For phospho-Etk, cells were pre-treated with 100 μM pervanadate for 15 min before harvest.

### MTT assay

Cells were seeded in 96-well plates and cultured overnight, followed by treatment with 0.1% DMSO, as the vehicle control, and CTA095, at the indicated concentrations for 72 h. Growth inhibition was measured using a 3-(4,5-Dimethylthiazol-2-yl)-2,5-diphenyltetrazolium bromide (MTT) assay (Roche Diagnostic, Mannheim, Germany).

### Flow cytometry

PC3 cells were treated with 0.1% DMSO (control) and CTA095 at the indicated concentrations for 24 h. Cell cycle arrest was determined by the incorporation of propidium iodide (Sigma-Aldrich) into permeabilized cells. Cells undergoing apoptosis were identified using an Annexin V-FITC kit (Abcam), following the manufacturer's instructions. The cells were analyzed using a Coulter Epics XL flow cytometer (Beckman Coulter, Miami, FL).

### Analysis of Caspase-3/7 and caspase 9 activities

For caspase 3/7 activities, PC3 cells were seeded at 5,000 cells/well in 96-well plate overnight. Then the cells were treated with 0–10 μM CTA095 for 24 h. Caspase-3/7 activities were measured using the Apo-ONE Homogeneous Caspase-3/7 Assay kit (Promega, Madison, WI) according to the manufacturer's instruction. For caspase 9 activity, PC3 cells were seeded in 10 mm tissue culture plate and grew to 50% confluence. Cells were then treated with 0–10 μM CTA095 for 24 h. Cells were harvested and caspase 9 activity was measured using a Western blot with an anti-caspase 9 antibody.

### Autophagy assay

PC3 cells were stably transfected with GFP-LC3 [16]. Cells were grown in a 6-well plate to 50% confluence and treated with 5 μM CTA095 for 24 h. Autophagy was visualized by GFP-LC3 “puncta” and immunoblot of Endogenous LC3 isoforms.

### HUVEC cell tube formation assay

HUVEC cells were seeded on mitrogel and treated with CTA095 (0 and 5 μM) for 6 h. Vascular tube formation was visualized using a microscope.

### PC3 cell “Wound healing” assay

PC3 cells were grown in 6-well plate to 60% confluency. Then wounds were made using a tip and treated with CTA095 (0 and 5 μM). Cell migration (wound healing) was visualized under microscope at the indicated times.

### Inhibition of PC3 xenograft tumor growth by CTA095nano (CAT095 formulated in nano-micelles)

This study was carried out in strict accordance with the recommendations in the Guide for the Care and Use of Laboratory Animals of the National Institutes of Health. The protocol was approved by the Institutional Animal Care and Use Committee (IACUC) of the University of California, Davis (Protocol Number: 16697). Briefly, 2×10^6^ PC3 cells were injected subcutaneously to the flanks of 5 week-old male nude mice. Tumor volume was measured by a caliper and calculated using the formula V = (L×W^2^)1/2. The tumors were grown to the indicated size and the mice were randomly divided into two groups (8 mice/group). The control group was treated with vehicle. The treatment group was treated with CTA095nano at 10 mg/kg twice a week via i.v. injection. The tumor size and body weight were measured once a week. The experiment was terminated when the tumor size of the control group reached humane endpoints by IACUC. Curves of tumor volumes verses duration were plotted for both groups.

### CTA095 overcomes Src inhibitor resistance in prostate cancer cells

PC3 and PC3-AZD20 (PC3 cell resistant to 20 μM AZD0530, which is from AstraZeneca through MTA with Dr. Chris Evans) cells were seeded at 2000 cells/well in 96 well plates overnight. The cells were treated with AZD0530 or CTA095 at the indicated concentrations. Cell viability was measured using an MTT assay after 72 h.

### CTA095 induces apoptosis in Src inhibitor resistant prostate cancer cells through Myc and BCL2 inhibition

PC3-AZD20 cells were seeded at 10^6^ cells/well in 6 well plates overnight. The cells were treated with AZD0530 or CTA095 at 10 μM. Apoptosis was analyzed using Annexin-V FITC apoptosis detection kit. The mRNA levels of Myc and BCL2 were measured using real-time PCR. pEtk, Etk, pSrc, Src, pStat3, Stat3, Myc and BCL2 levels were measured using the corresponding antibodies through a Western blot.

### Statistics

A one-way ANOVA was used in combination with a Tukey test for pair wise comparison. P values less than 0.05 were considered significant.

## Results

### CTA095 as a dual inhibitor against Etk and Src tyrosine kinases

**Figure 1:**
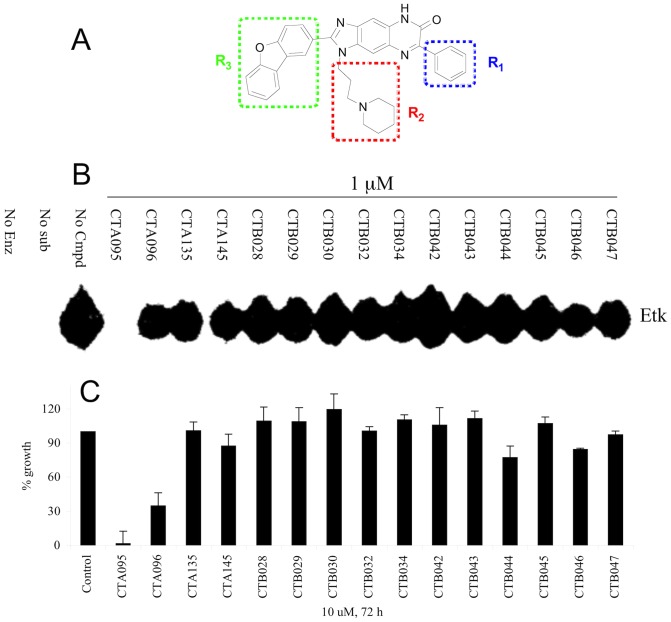
CTA095 inhibits Etk activity and PC3 cell growth. Chemical structure of CTA095 (A), identification of CTA095 as a potent Etk inhibitor (B) and its cytotoxicity to PC3 cells (C). For Etk inhibition, purified Etk (20 nM), the corresponding compounds (1 μM), and the peptide substrate (YIYGSFK) were incubated with ^33^P-ATP in a kinase reaction. The resulting product was analyzed on a TLC plate. For growth inhibition, PC3 cells were seeded at 5,000 cells/well in 96-well plate overnight and treated with the corresponding compounds (10 μM) The cell viability was measured using MTT assay after 72 h. Columns, mean; bars, standard deviation, n = 3.

Through screening a 9,600-diversity combinatorial solution phase small molecule library, hit compounds with inhibitory activities against Etk were discovered. Subsequent structure-activity-relationship studies led to the identification of CTA095 (Figure 1A). Compared with other CT compounds with a fused three-ring core structure identical to CTA095, CTA095 showed significant Etk inhibition (Figure 1B). Interestingly, there was a strong correlation between Etk inhibition and PC3 growth inhibition (Figure 1C). This data suggests that Etk may be the target responsible for the growth inhibition observed for PC3 cells.

**Figure 2:**
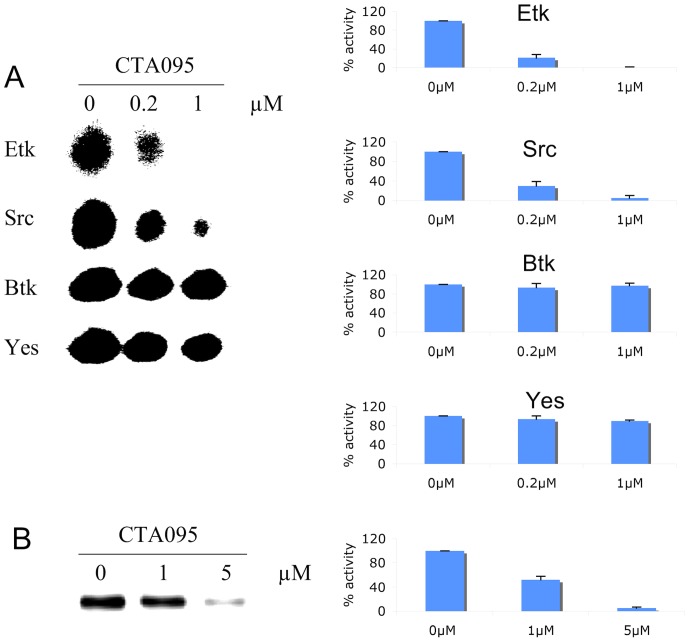
CTA095 is a potent Etk and Src dual inhibitor. The potency of CTA095 to Etk, Src, Btk and Yes was measured using TLC (A) to identify ^33^P-phosphorylated peptide substrate. Purified TKs (20 nM), CTA095 (0, 0.2, and 1 μM), and the peptide substrate (YIYGSFK) were incubated with ^33^P-ATP in a kinase reaction. The resulting product was analyzed on a TLC plate. The effect of CTA095 to Etk autophosphorylation (B) was further examined by incubation of Etk with CTA095 at the indicated concentrations in the presence of ^33^P-ATP, the radioactivity of Etk was measured using radiosensitive film. The intensity of the radioactive spot was measured using densitometer. Columns, mean; bars, standard deviation, n = 3.

Having isolated a small molecule that inhibits Etk, the next logical step was to determine its specificity for this enzyme. To accomplish this, purified Etk, Btk, Src and Yes were incubated in a kinase reaction buffer with CTA095 (0–1 μM) in the presence of ^33^P-labeled ATP and a peptide (YIYGSFK), previously shown to be an excellent substrate for both BTK and Src family kinases. The kinase activity was measured using TLC technique. This study revealed that CTA095 was a potent inhibitor for Etk, with an IC_50_ of approximately 60 nM (Figure 2A). Inhibition was observed in a concentration dependent manner. Moreover, CTA095 could also inhibit Src (IC_50_ ≈ 120 nM). However, Btk and Yes were significantly more resistant to CTA095 inhibition with IC_50_ greater than 10 μM (Figure 2A, Figure S2).

The Btk family of non-receptor tyrosine kinases is characterized by the presence of an autophosphorylation site within the non-catalytic Src homology 3 (SH3) domain. Thus, it was also important to determine the ability of CTA095 to inhibit Etk autophosphorylation. Therefore, an *in vitro* Etk autophosphorylation assay was established in which purified Etk was mixed with CTA095 in the presence of ^33^P-ATP. After 30 min, the reaction was terminated, and the samples were loaded onto an SDS-polyacrylamide gel for electrophoresis. After drying, the gel was analyzed with a phosphoimager. Figure 2B reveals that CTA095 was able to inhibit Etk autophosphorylation in a concentration dependent manner.

In addition to the Btk family tyrosine kinases, the inhibitory activity of CTA095 to other kinases, including Lyn, Axl, Mer, EGFR, and Abl, was investigated using a TLC assay. As shown in Table 1, CTA095 appears to have strong reactivity toward Etk and Src, much higher than that of any other kinases tested.

**Table 1:** Kinase inhibition profile of CTA095.

Kinase	IC_50_ (μM)
Etk	0.06
Src	0.12
Btk	27
Yes	>10
Itk	>10
Lyn	>10
Axl	>10
Mer	>10
EGFR	>10
Abl	>10

### CTA095 inhibits Etk through binding of its ATP binding region

**Figure 3:**
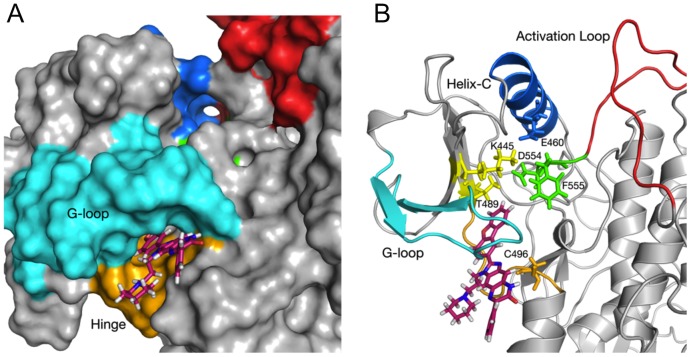
Molecular Modeling of CTA095-Etk binding. (A) Surface view of Etk docked to CTA095 after 20 ns of MD minimization and relaxation. R_3_ and the three-ring core docked deeper between Glycine-rich loop region (cyan) and hinge region (orange), R_1_ and R_2_ are solvent exposed. Blue: Helix C; Red: Activation Loop; Green: DFG motif (554–556); Cyan: Glycine-rich loop; Orange; Hinge Region. (B) Cartoon representation showing predicted interactions of CTA095 with the gatekeeper Thr489, DFG (554–556) motif, and Cys496. CTA095 binding stabilizes Phe555 in ‘out’ configuration, and affects the active state salt bridge formation between Lys445 and Glu460. Blue: Helix C; Red: Activation Loop; Green: DFG motif (554–556); Cyan: Glycine-rich loop; Orange; Hinge Region. Figures were generated using PyMol.

To explore the putative mechanism responsible for Etk inhibition by CTA095, molecular docking and dynamics studies were performed. These studies predict that CTA095 interacts with the back-pocket of the ATP binding region. This binding pocket is formed by the residues in the Glycine rich loop, ‘gatekeeper’ T489, and the hinge region (Figure 3A). The R_3_ group of CTA095 interacts with the gatekeeper molecule Thr489, and also stabilizes the Phe555 of DFG motif in an inactive ‘out’ position (Figure 3B). Moreover, the three-ring core group of the compound interacts with the Cys496, which is very unique to the Etk. Only 8 kinases out of the 491 kinases that were analyzed in a previous study [29], [30] show Threonine and Cysteine at these positions. Thus CTA095's interaction with both Thr489 and Cys496 may provide it with unique kinase selectivity. We also used LigPlot+ to predict hydrogen bonding and/or hydrophobic interactions in the binding pocket, and our results showed that multiple hydrophobic interactions are responsible for the CTA095-Etk binding (Figure S3A and S3B). Side chains that putatively interact with CTA095 are shown in Figure S3C. Analysis of the molecular dynamics trajectories also show that the R_3_ group and the three-ring core interact strongly and stably with the side chains in the binding pocket, while R_1_ and parts of R_2_ are solvent exposed, and may serve as targets for further improvement of the CTA095 binding and specificity (Movie S1).

Unlike ETK-CTA095 binding, Src kinase shows binding with CTA095 in the active site pocket formed by the N-lobe, C-lobe and the activation loop. CTA095 in its docked position spans the residues Asp404 and Asn 391, which are both important for Mg^2+^ and ATP binding. CTA095 also putatively interacts with the functionally important Tyr416 residue, which is part of the activation loop region (Figure S4). Overall, we believe that CTA095 blocks the ATP binding pocket in Src kinase, and inhibits ATP binding in Etk by inducing conformational changes via the back-pocket.

### CTA095 inhibits the phosphorylation of Etk, Src and the downstream signals Stat3 and Akt in prostate cancer cells

**Figure 4:**
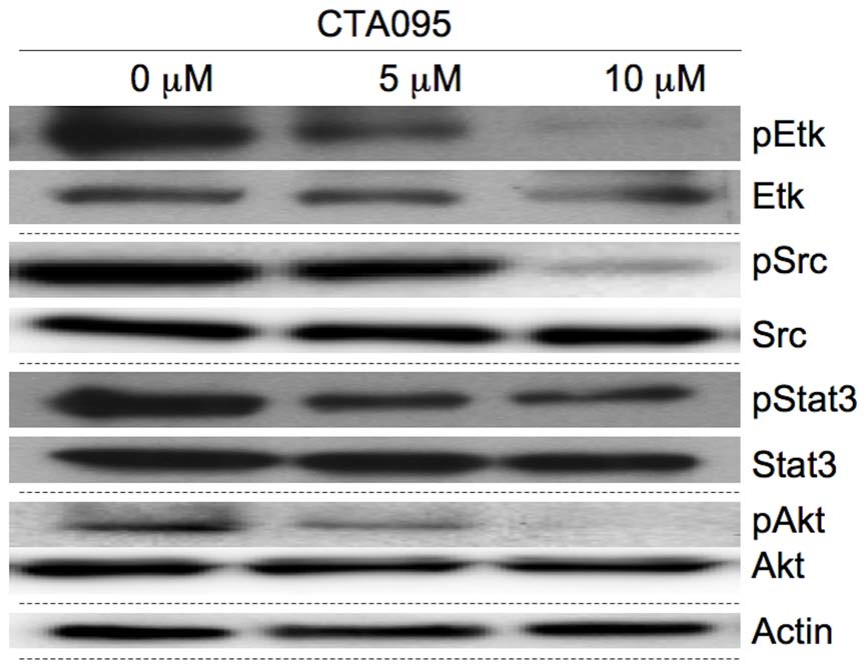
Inhibition of cell signaling in PC3 cells following treatment with CTA095. Cells were grown in 10 mm plate to 50% confluence and treated with CTA095. Cells were harvested after 24 h. pEtk, Etk, pSrc, Src, pStat3, Stat3, pAkt and Akt levels were measured using the corresponding antibodies by Western blot. One of three similar experiments depicted.

The inhibitory activity of CTA095 against phosphorylation of Etk in intact cells was examined by Western blot. Etk, as well as Src phosphorylation in PC3-Etk (PC3 cells stably transfected with Etk), cells were significantly inhibited at 5 μM and 10 μM. The Src inhibition is likely to result from both direct inhibition by CTA095, as well as the diminished activity Etk, which activates Src. A selective target for Etk and Src is STAT3, whose phosphorylation is also inhibited by CTA095. Akt is another important downstream effector of Etk, and its phosphorylation was inhibited by CTA095 (Figure 4).

### CTA095 preferentially inhibits the growth of malignant prostate cells

**Figure 5:**
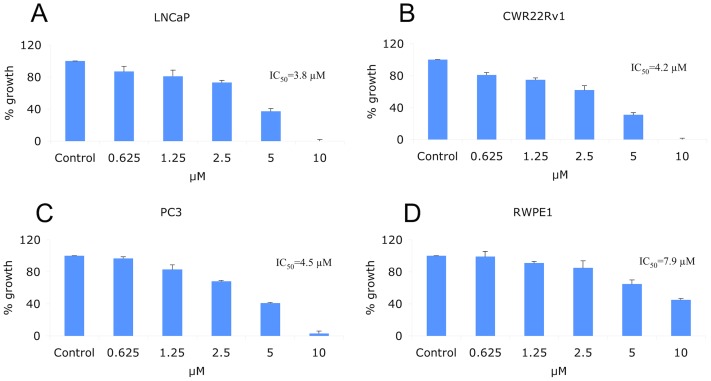
Growth Inhibition of CTA095 to LNCAP, CWR22Rv1, PC3 prostate cancer and normal prostate (RWPE1) cells. Cells were seeded at 5,000 cells/well in 96-well plate overnight and treated with CTA095 at the indicated concentrations. The cell viability was measured using MTT assay after 72 h. dots, mean; bars, standard deviation, n = 3.

To determine the effect of CTA095 on proliferation, a panel of cancer cell lines including LNCAP, CWR22Rv1, PC3 and the normal prostate cell RWPE1 were incubated with CTA095 and their proliferation was measured using the MTT assay. CTA095 was effective in inhibiting the growth of prostate cancer cells (LNCAP, CWR22Rv1 and PC3), while the immortalized normal prostate cell RWPE1 was more resistant to CTA095 (Figure 5), likely due to the “addiction” to Etk signal by prostate cancer cells, as shown previously [11].

### CTA095 induces autophagy in prostate cancer cells

**Figure 6:**
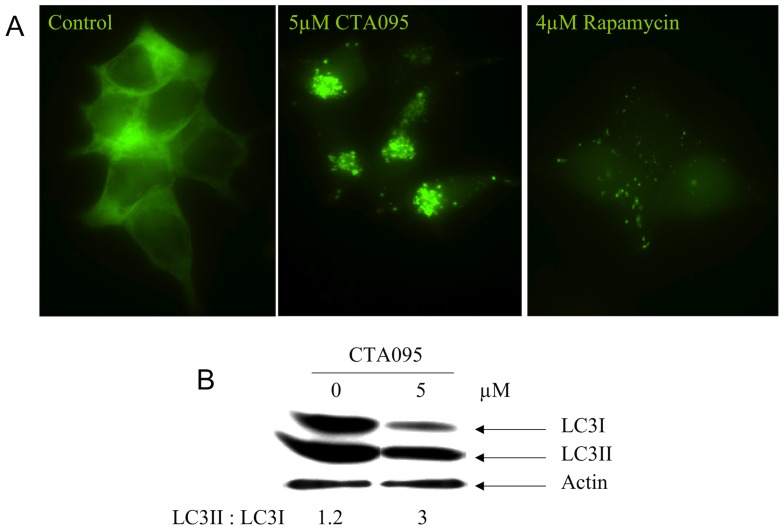
Induction of autophagy in PC3 cells by CTA095. Cells were grown in 6-well plate to 50% confluence and treated with CTA095. Autophagy was visualized by GFP-LC3 “puncta” (A) and immunoblot of Endogenous LC3 isoforms (B). All experiments were carried out 24 h after treatment.

Previously we showed that the Src inhibitor AZD0530 induces autophagy in prostate cancer cells, which contributes to apoptosis resistance and diminishes the efficacy of the Src inhibitor [16]. To determine whether CTA095 can trigger autophagy, PC3 cells stably transfected with GFP-LC3 were treated with CTA095, then examined under fluorescence microscopy. After 24 h treatment with CTA095, these cells yielded extensive distinct “puncta” autophagosome morphology, whereas vehicle treatment did not (Figure 6A). The ability of CTA095 to induce autophagy in PC3 cells was further confirmed by the conversion of endogenous LC3-I to LC3-II forms (Figure 6B). Thus, like the Src tyrosine kinase inhibitor, inhibition of Etk also induces autophagy.

### CTA095 induces apoptosis in prostate cancer cells

**Figure 7:**
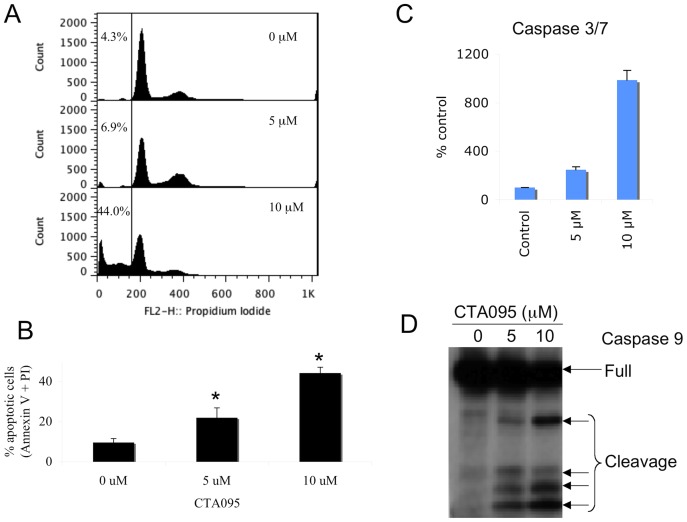
Induction of apoptosis of PC3 cells following treatment with CTA095. PC3 cells were seeded at 10^6^ cells/ml (2 ml) in a 6-well plate overnight and then treated with CTA095 at the indicated concentrations for 24 h. Cell cycle arrest was analyzed using PI staining (A). Apoptosis was analyzed using Annexin-V FITC apoptosis detection kit (B). Caspase 9 activation was measured using western blot (D). For caspase 3/7 activity, PC3 cells were seeded at 5000 cells/well in 96 well plate overnight and treated with CTA095 at 0–10 μM for 24 h. Caspase-3/7 activities were measured using the Apo-ONE Homogeneous Caspase-3/7 Assay kit (Promega, Madison, WI) according to the manufacturer's instruction. Columns, mean; bars, standard deviation, n = 3. 5 μM and 10 μM are significantly different from 0 μM (*, p<0.05, one-way ANOVA with Tukey test for pair wise comparison).

To determine whether the growth inhibition induced by CTA095 on PC3 cells was due to apoptosis, flow cytometric analysis was carried out. Following treatment with CTA095 for 24 h, a dose dependent accumulation of a “sub-G1” fraction was observed using PI staining (Figure 7A). Data based on Annexin-V reactivity also indicated a dose-dependent increase of apoptosis of PC3 cells following treatment with CTA095 (Figure 7B). Further investigation indicated that treatment of PC3 cells with CTA095 resulted in a dose dependent activation of caspase3/7 (Figure 7C) and caspase 9 (Figure 7D). Thus, in contrast to the Src inhibitor AZD05350, this Etk inhibitor induces a significant level of apoptosis, despite its stimulation of the autophagy pathway. More interestingly, 293 cells (Etk negative) are resistant to induce apoptosis by CTA095, indicating the specificity of this compound to Etk (Figure S5). As will be discussed later, Etk directly controls the survival pathway, the absence of which apparently can override the protective effect of autophagy. As a result, CTA095 induced apoptosis can be further enhanced by an autophagy blockade (see below).

### CTA095 inhibits HUVEC cell tube formation and prostate cancer cell migration

**Figure 8:**
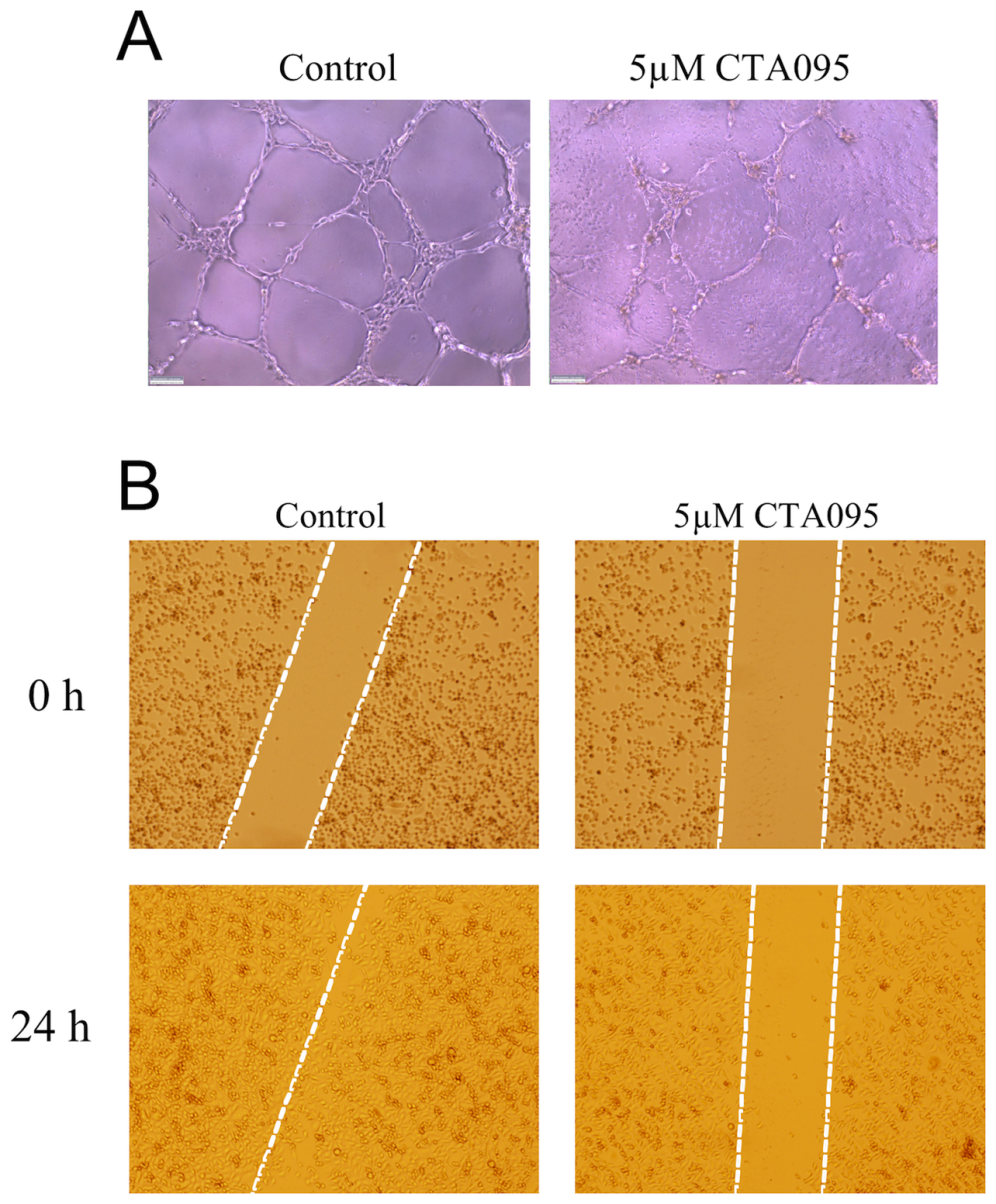
Inhibition of tube formation and cell migration by CTA095. Inhibition of vascular tube formation of HUVEC endothelial cells (A) and inhibition of migration (wound healing) of PC3 human prostate cancer cells (B) by CTA095. (A) HUVEC cells were seed on mitrogel and treated with CTA095 (0 and 5 μM) for 6 h. Vascular tube formation was visualized using microscope. (B) PC3 cells were grown in 6-well plate to 60% confluency. Then wounds were made and treated with CTA095 (0 and 5 μM). Cell migration (wound healing) was visualized under microscope at the indicated times.

Etk is highly expressed in endothelial cells and shown to be involved in angiogenesis [31]. To investigate the ability of CTA095 to inhibit angiogenesis, vascular tube formation of HUVEC endothelial cells following treatment with CTA095 was examined. As expected, tube formation of HUVEC cells was disrupted by CTA095 (Figure 8A). To explore the ability of CTA095 to inhibit cell migration, “wound healing” of PC3 cells was measured following treatment with CTA095. As shown in Figure 8B, “wound healing” of PC3 cells was greatly inhibited by CTA095 after 48 h. These results suggest that CTA095 has the ability not only to suppress prostate cancer cell growth, but also to reduce angiogenesis.

### CTA095 as a chemo-sensitizer

**Figure 9:**
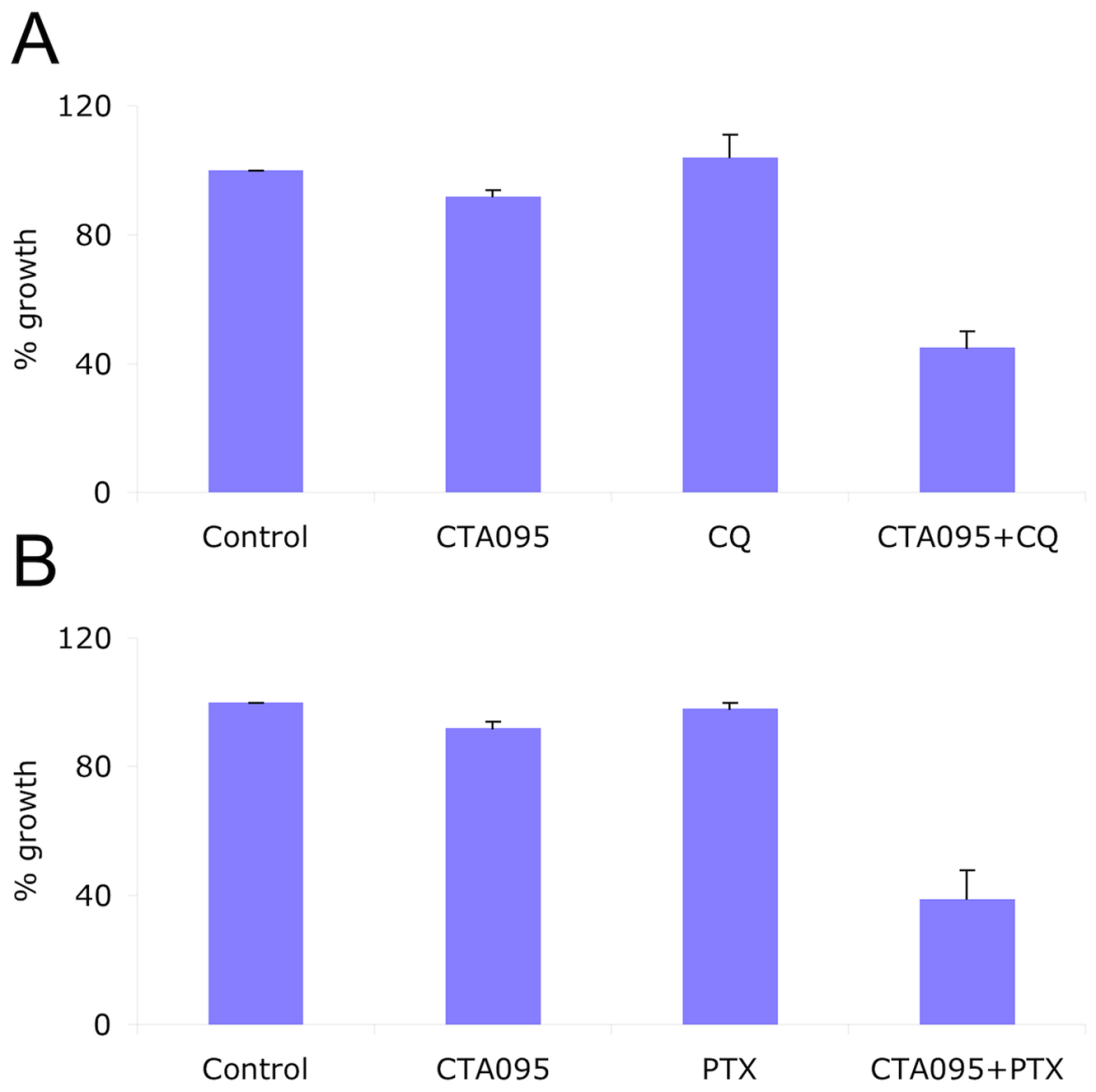
CTA095 as a chemo sensitizer. Growth Inhibition of CTA095 and in combination with 10 μM chloroquine (CQ), or 2 ng/ml paclitaxel (PTX) to PC3 human prostate cancer cells. Cells were seeded at 5,000 cells/well in 96-well plate overnight and pretreated with the corresponding co-treatments for 1h, then treated with 2.5 μM CTA095. The cell viability was measured using MTT assay after 72 h. Columns, mean; bars, standard deviation, n = 3.

Our initial studies indicated that CTA095 has good cytotoxicity toward a panel of prostate cancer cells. To examine whether Etk and Src dual inhibitor works effectively as a chemo sensitizer, PC3 cells were co-treated with CTA095 and paclitaxel (PTX) (2ng/ml), or the autophagy inhibitor chloroquine (CQ) (10 μM). Growth inhibition was determined using an MTT assay after 72 h. Interestingly, a synergistic effect of CTA095 with PTX or CQ to prostate cancer cells was observed (Figure 9). This suggests that CTA095 is a chemo sensitizer, and blocking of autophagy by CQ promotes CTA095 induced cell death.

### CTA095nano inhibits PC3 xenograft tumor growth in vivo

**Figure 10:**
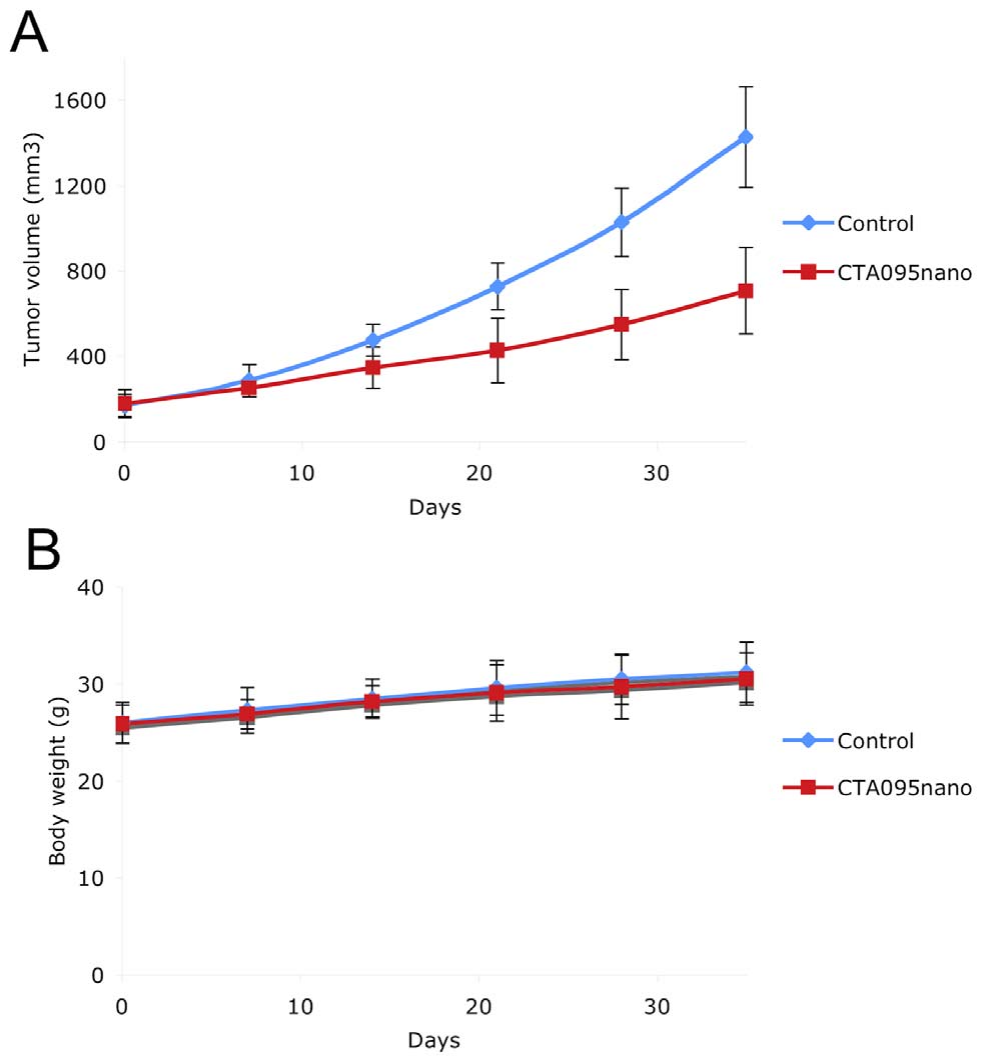
Inhibition of PC3 xenograft tumor growth by CTA095nano. (CAT095 formulated in nano-micelles.) 2×10^6^ PC3 cells were injected subcutaneously to nude mice. The tumors were grown the indicated size and the mice were randomly divided into two groups (8 mice/group). The control group was treated with vehicle. The treatment group was treated with CTA095nano at 10 mg/kg twice a week with iv injection. The tumor size (A) and body weight (B) were measured once a week. Marks, mean; bars, sd. n = 8.

Given the *in vitro* activity of CTA095 against prostate cancer cells, it is important to validate these results *in vivo*. Since CTA095 is highly insoluble in water, we formulated CTA095 into nano micelles. This micelle was developed in our lab and has been used successfully to formulate hydrophobic drugs such as paclitaxel and vincristine [32], [33]. As shown in Figure 10, CTA095nano significantly prevented PC3 xenograft tumor growth at 10 mg/kg (twice a week, intra-venous injection) without significant toxicity.

### CTA095 overcomes Src inhibitor resistance in prostate cancer cells

**Figure 11:**
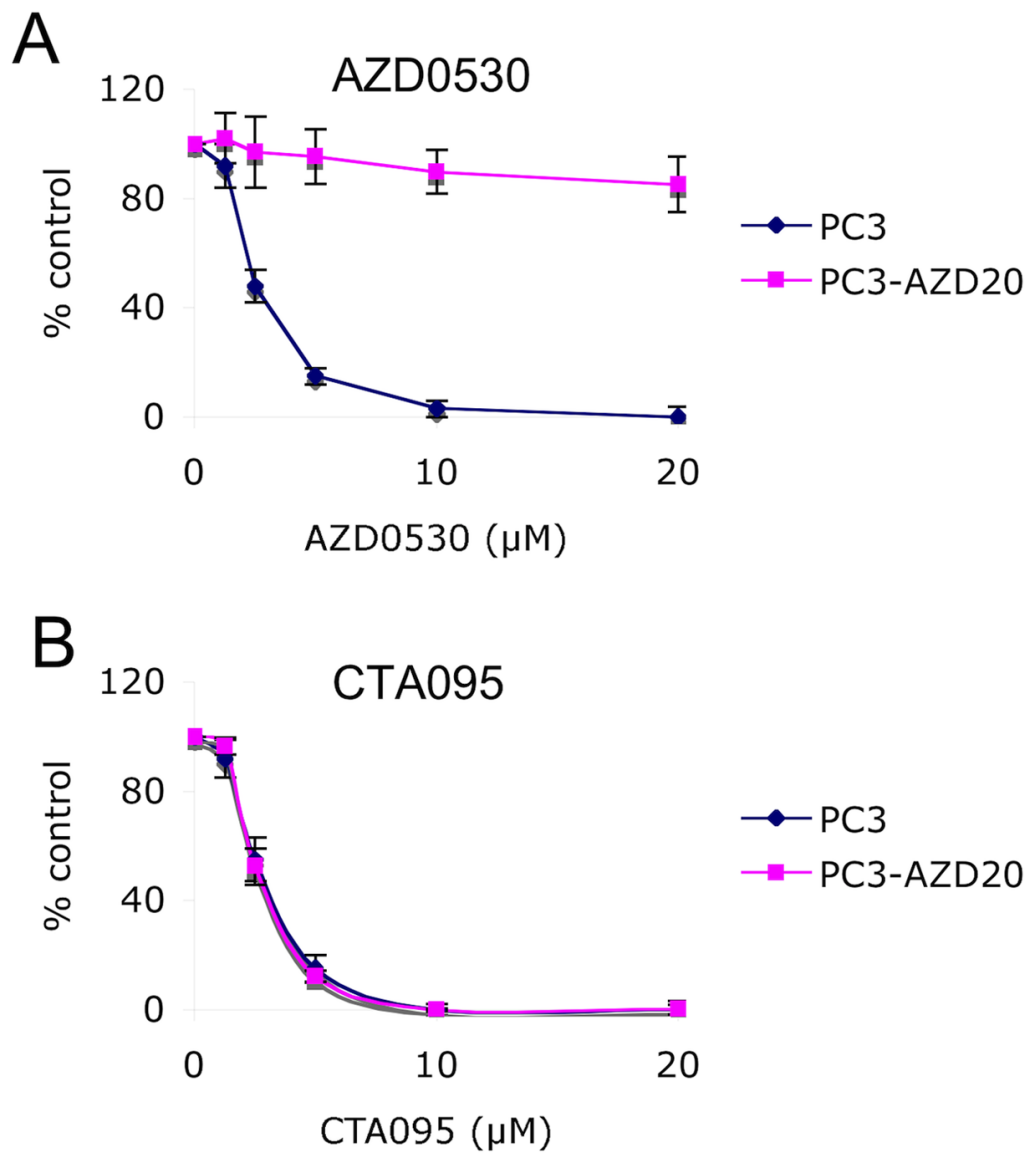
CTA095 overcomes Src inhibitor resistance in prostate cancer cells. PC3 and PC3-AZD20 (PC3 cell resistant to 20 μM AZD0530) cells were seeded at 2000 cells/well in 96 well plates overnight. The cells were treated with AZD0530 or CTA095 at the indicated concentrations. Cell viability was measured using MTT assay after 72 h. Symbols, mean; bars, standard deviation, n = 3.

**Figure 12:**
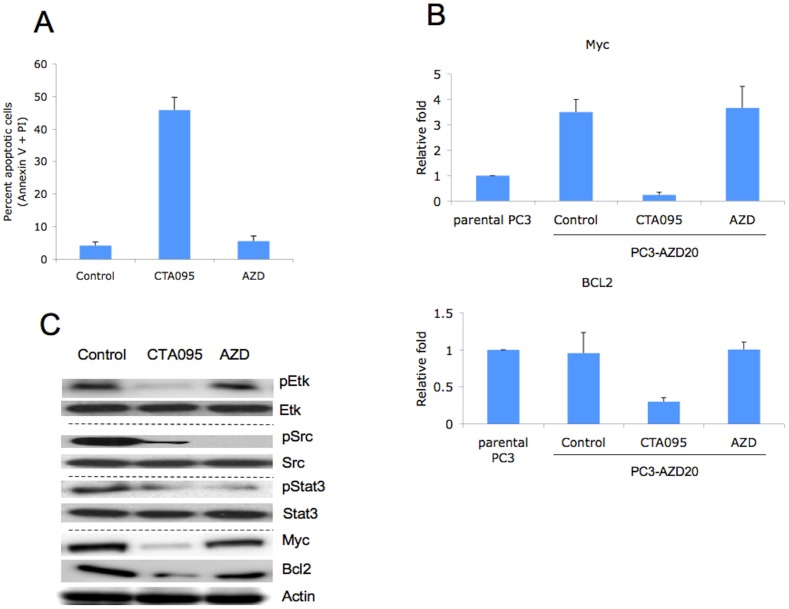
CTA095 induces apoptosis in Src inhibitor resistant prostate cancer cells through Myc and BCL2 inhibition. PC3-AZD20 (PC3 cell resistant to 20 μM AZD0530) cells were seeded at 10^6^ cells/well in 6 well plates overnight. The cells were treated with AZD0530 or CTA095 at 10 μM. Apoptosis was analyzed using Annexin-V FITC apoptosis detection kit (A). The mRNA levels of Myc and BCL2 were measured using real-time PCR (B). pEtk, Etk, pSrc, Src, pStat3, Stat3, Myc and BCL2 levels were measured using the corresponding antibodies through Western blot (C). Columns, mean; bars, standard deviation, n = 3.

Since CTA095 induces a significant amount of caspase activation and apoptosis in prostate cancer cells, which is different from the Src inhibitor AZD0530, we asked whether CTA095 could enhance the efficacy of Src inhibitors, and whether it could overcome prostate cancer cell resistance developed toward this Src inhibitor. A mixed population of AZD0530 resistant cells, PC3-AZD20, was developed by long-term culture of PC3 cells with gradually increasing AZD0530 concentrations up to 20 μM. PC3-AZD20 cells were resistant to 20 μM AZD0530 (Figure 11A), while highly sensitive to CTA095, with an IC50 almost identical to that of the parental PC3 cells (Figure 11B). Massive apoptosis was induced in PC3-AZD20 (PC3 cell resistant to 20 μM AZD0530) cells following treatment with CTA095, but not with treatment of AZD0530 (Figure 12A). These results suggest that the antiapoptotic activities conferred by Etk are involved in the resistance to Src inhibitor and CTA095, as a dual inhibitor of Etk and Src, counteract both anti-apoptosis effects.

### CTA095 induces apoptosis in Src inhibitor resistant prostate cancer cells through Myc and BCL2 inhibition

To explore the molecular basis of Src inhibitor resistance in PC3-AZD20, cDNA microarrays were conducted for both PC3-AZD20 and the parental PC3 cell line. Among the genes up-regulated are c-myc and bcl-2. RT-PCR studies indicated that Myc, as well as BCL2, were significantly down regulated in PC3-AZD20 cells following treatment with CTA095 (Figure 12B), indicating that this down regulation may contribute to the induction of apoptosis in Src inhibitor resistant prostate cancer cells. Interestingly, a Western blot indicated that pSrc and pSTAT3, but not c-myc and Bcl-2 were decreased in PC3-AZD20 cells following treatment with AZD0530. pEtk, pSrc, pSTAT3, Myc and BCL2 were all down regulated following treatment with CTA095, implying that pEtk inhibition may lead to down regulation of Myc and BCL2 and subsequent apoptosis (Figure 12C). The more detailed signaling pathways between this link remains to be further elucidated.

## Discussion

Tyrosine kinases have become important targets for drug development. Powerful combinatorial chemistry approaches and high throughput screening assays have led to successful identification of many kinase inhibitors [34]–[37]. AZD0530, an Src kinase inhibitor, has been to shown to be effective in inhibiting prostate cancer cell bone metastasis in animal models, but AZD0530 lacks the activity to induce apoptosis. Previous work in our lab indicated that Etk is complexed with Src and FAK, and that it plays an important role in apoptosis, angiogenesis, and metastasis of prostate cancer cells. While Src signals primarily regulate cell growth, the Etk and FAK are involved in cell migration. In addition, Etk interacts with p53 and controls the apoptosis pathway. Inhibition of Src leads to cell cycle arrest and reduction of migration and inhibition. Dual inhibition of Src and Etk are expected to inhibit growth, migration, and survival of the cells. To the best of our knowledge, this is the first selective Etk/Src inhibitor reported to date.

**Figure 13:**
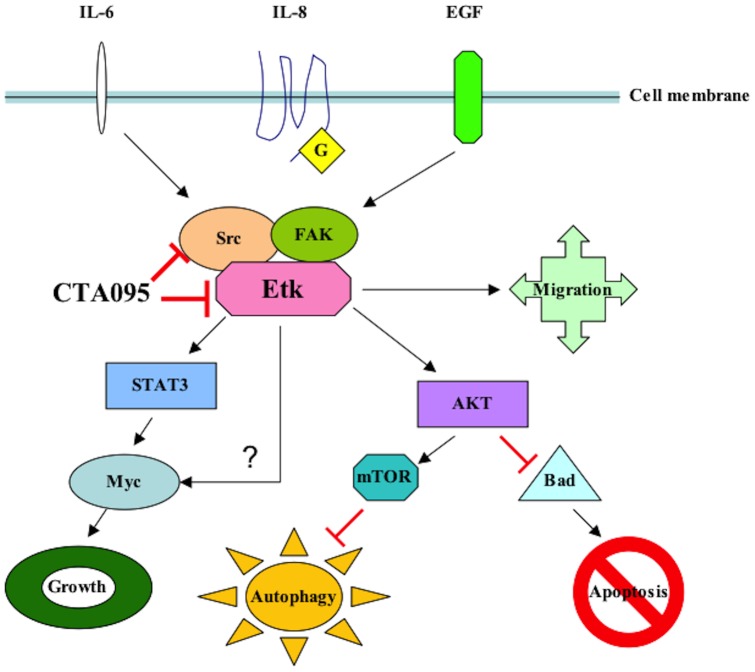
Proposed signal pathways of CTA095 in prostate cancer cells.

CTA095 belongs to a novel class of kinase inhibitors with a chemical structure distinct from other known TKIs. This inhibitor is most potent in inhibiting Etk followed by Src, but it is not a potent inhibitor for other Tec and Src family kinases such as Btk, Yes and Lyn. Treatment of PC3 cells with CTA095 resulted in the decrease of phoshorylation of Etk and Src, as well as the downstream signals Stat3 and Akt. CTA095 has little effect on the survival of the immortalized normal prostate cell RWPE1, suggesting that the growth and survival of prostate cancer cells are more dependent on Etk and Src signals, consistent with the observed overexpression of Etk in prostate cancer tissues and the development of PIN phenotype in transgenic animals carrying overexpressed Etk [11]. Etk interacts with and inactivates p53, and overexpression of Etk in prostate cancer cells confers resistance to androgen deprivation [38] and photodynamic therapy [3]. On the other hand, truncated Etk, due to caspase cleavage, induces apoptosis [39]. This data taken together suggests that Etk is a strong regulator of apoptosis. At the same time, CTA095 was found to induce autophagy in human prostate cancer cells. Autophagy is generally regarded as a pro-survival mechanism, where wasted proteins and retired organs are degraded to regenerate energy during stress conditions. It is inhibited by energy sensor mTOR, a target of rapamycin (RPM). Excessive autophagy, under special conditions, however, can lead to programmed cell death (type II). Previously, we reported that autophagy induced by Src inhibitor is the underlying cause of apoptosis-resistance of the treated cells [16]. These cells are growth arrested, but do not undergo apoptosis. Blocking autophagy by siRNA targeting of autophagy components, or the simultaneous application of CQ, an inhibitor of autophagy flow, results in massive cell death. It is interesting that, like the Src inhibitor, CTA095 induced autophagy is likely due to its inhibition of mTOR activation. However, unlike the Src inhibitor, CTA095 inhibits both Etk and Src, and can overcome the apoptosis resistance induced by autophagy. This data suggests that the absence of Etk proactively turns on the apoptosis pathway, which cannot be completely overridden by the restoring act of autophagy. The finding that the autophagy inhibitor CQ further enhances the apoptosis-inducing effect of CTA095 suggests that autophagy contributes partially to the survival of CTA095 treated cells. Alternatively, CQ is known to have other cellular effects including induction of p53 and intercalation of DNA, which may contribute additional toxicity to the treated cells. Curiously, we found that the autophagy inducer RPM also synergizes with CTA095, suggesting excessive autophagy may contribute additional means to kill prostate cancer cells. CTA095 was also a chemo-sensitizer and showed synergistic effect with PTX, indicating its role in combination therapy for prostate cancer. In addition to these findings, CTA095 was found to inhibit HUVEC cell tube formation and “wound healing” of prostate cancer cells, implying its role in inhibition of angiogenesis and metastasis of human prostate cancer cells (Figure 13).

More interestingly, simultaneously targeting of Etk and Src by CTA095 overcomes the Src inhibitor resistance in prostate cancer cells. The Src inhibitor AZD0530 has a good effect on inhibition of bone metastasis of prostate cancer, but lacks the ability to induce apoptosis in prostate cancer cells. Dual inhibition of Etk and Src could overcome this disadvantage and induce massive apoptosis in Src inhibitor-resistant prostate cancer cells. This is likely through the inhibition of Etk and down regulation of Myc and BCL2. The more detailed signaling pathways between the link remains to be further investigated.

In summary, we have identified an Etk and Src dual inhibitor, CTA095, with good selectivity toward prostate cancer cells. This inhibitor could overcome Src inhibitor resistance and induce apoptosis in Src inhibitor-resistant prostate cancer cells. This study indicates that Etk and Src dual inhibition holds exceptional promise as a novel treatment strategy for prostate cancer.

## Supporting Information

Figure S1 **Synthetic scheme of CTA095.**
(TIF)

Figure S2 **Inhibition of Btk by CTA095.** Purified Btk (20 nM), CTA095 (0–100 μM), ATP (500 μM) and the peptide substrate (YIYGSFK) were incubated in a kinase reaction. The kinase activity was measured using Kinase-Glo assay kit (Promega Inc.) following the manufacture's instruction. Columns, mean; bars, standard deviation, n = 3.
(TIF)

Figure S3 **Predicted hydrophobic and hydrophilic interactions between Etk and CTA095.** Interactions between CTA095 and Etk as predicted using LigPlot+ from MD trajectories after (A) 10 ns of relaxation (B) 20 ns of relaxation. Red dashed arrows: Hydrophobic interactions; Green: Hydrogen bonding. (C) Putative interactions between CTA095 and ETK residue side chains colored according to charge properties Green: Polar; Orange: Non-Polar; Blue: Basic.
(TIF)

Figure S4 **Molecular modeling of CTA095-Src binding.**
(TIF)

Figure S5 **Induction of apoptosis of 293 cells following treatment with CTA095.** 293 cells were seeded at 10^6^ cells/ml (2 ml) in a 6-well plate overnight and then treated with CTA095 at the indicated concentrations for 24 h. Apoptosis was analyzed using Annexin-V FITC apoptosis detection kit. Columns, mean; bars, standard deviation, n = 3.
(TIF)

Movie S1 **Molecular Dynamics Trajectory of Etk bound to CTA095.** Movie shows interactions between CTA095 (in licorice representation) and side chains of Thr489 (gatekeeper molecule), Asp554, Cys480, Phe555, Lys445 and Glu460 (shown as sticks). Lime Green: Helix C; Light Blue: Activation Loop; Orange: Glycine-rich loop; Movie generated using VMD.
(MPG)
