# Supplementary figures and images for: CTA095, a Novel Etk and Src Dual Inhibitor, Induces Apoptosis in Prostate Cancer Cells and Overcomes Resistance to Src Inhibitors
Source: PLoS One. 2013 Aug 15;8(8):e70910. doi: 10.1371/journal.pone.0070910 (PMC3744530; doi:10.1371/journal.pone.0070910)

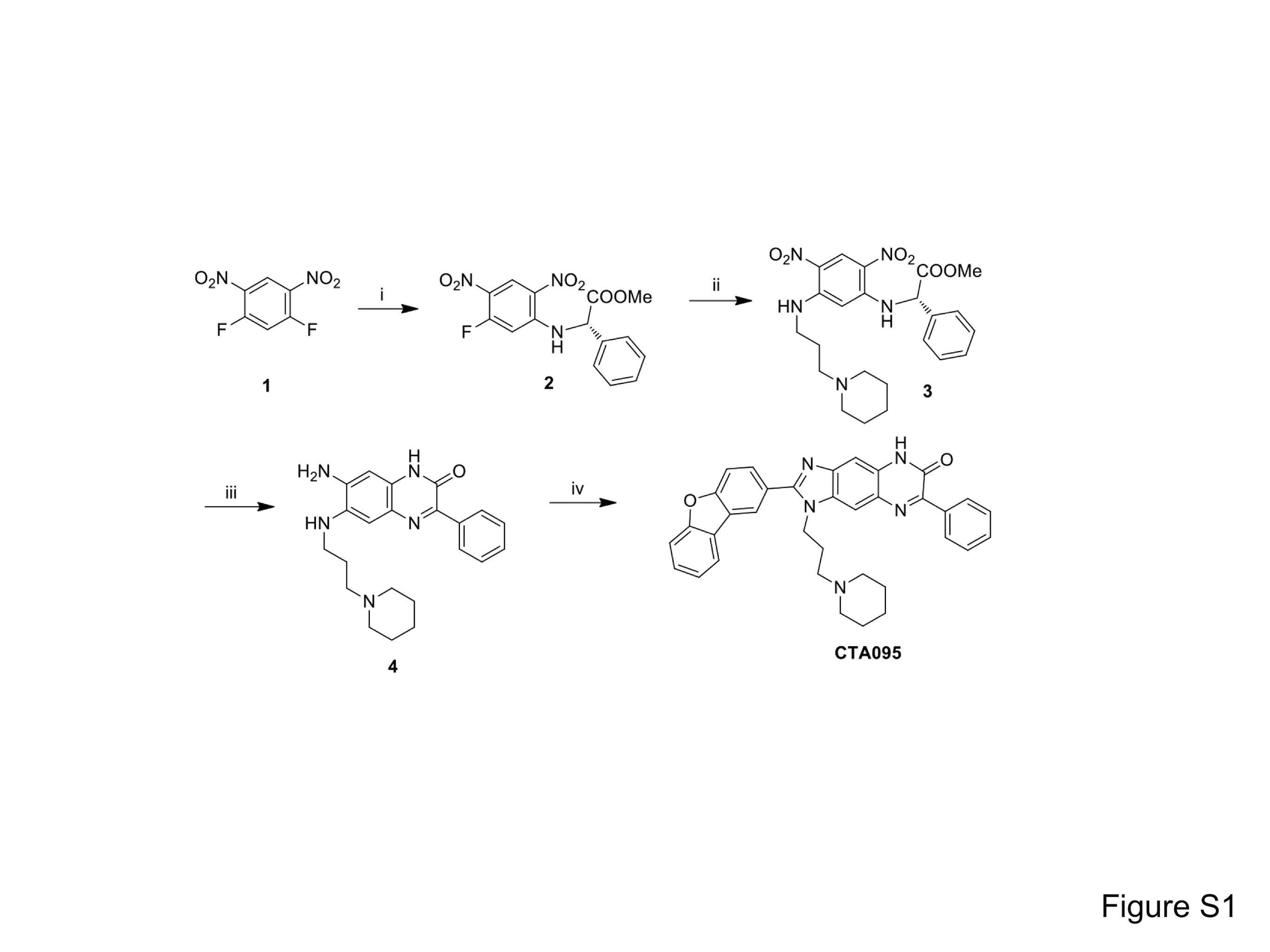

Supplement: Figure S1 — Synthetic scheme of CTA095. (TIF) [file pone.0070910.s001.tif]

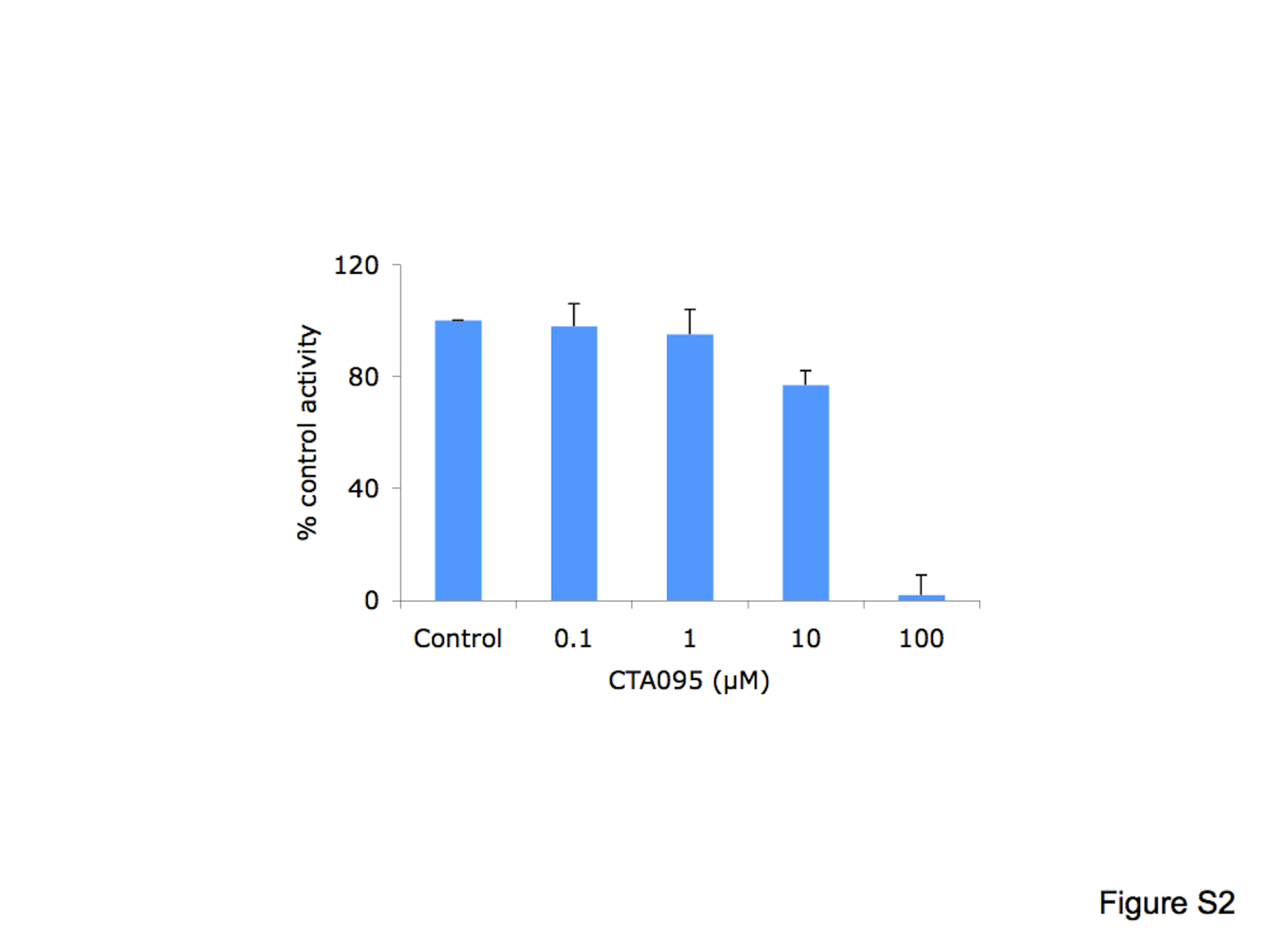

Supplement: Figure S2 — Inhibition of Btk by CTA095. Purified Btk (20 nM), CTA095 (0–100 μM), ATP (500 μM) and the peptide substrate (YIYGSFK) were incubated in a kinase reaction. The kinase activity was measured using Kinase-Glo assay kit (Promega Inc.) following the manufacture's instruction. Columns, mean; bars, standard deviation, n = 3. (TIF) [file pone.0070910.s002.tif]

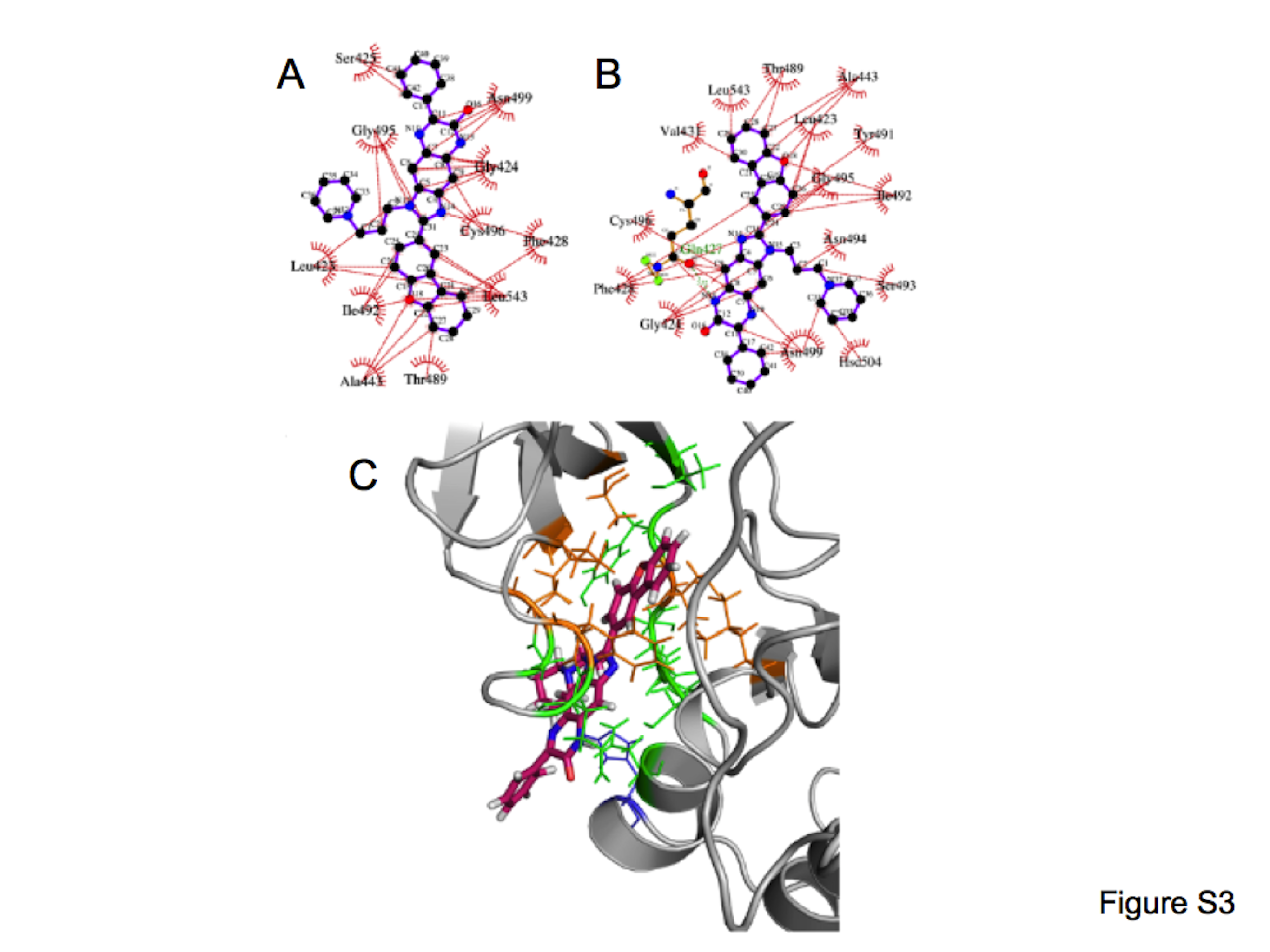

Supplement: Figure S3 — Predicted hydrophobic and hydrophilic interactions between Etk and CTA095. Interactions between CTA095 and Etk as predicted using LigPlot+ from MD trajectories after (A) 10 ns of relaxation (B) 20 ns of relaxation. Red dashed arrows: Hydrophobic interactions; Green: Hydrogen bonding. (C) Putative interactions between CTA095 and ETK residue side chains colored according to charge properties Green: Polar; Orange: Non-Polar; Blue: Basic. (TIF) [file pone.0070910.s003.tif]

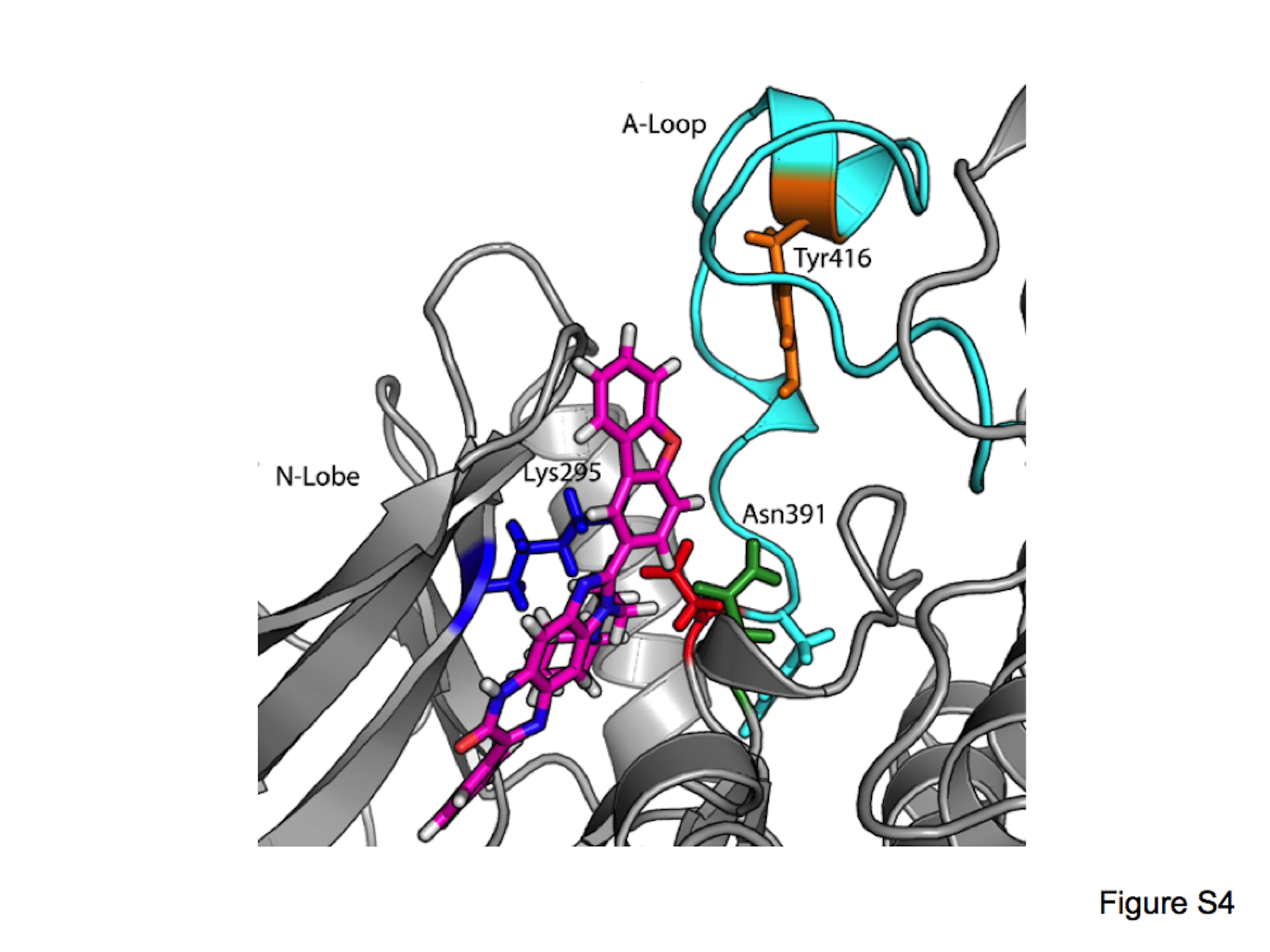

Supplement: Figure S4 — Molecular modeling of CTA095-Src binding. (TIF) [file pone.0070910.s004.tif]

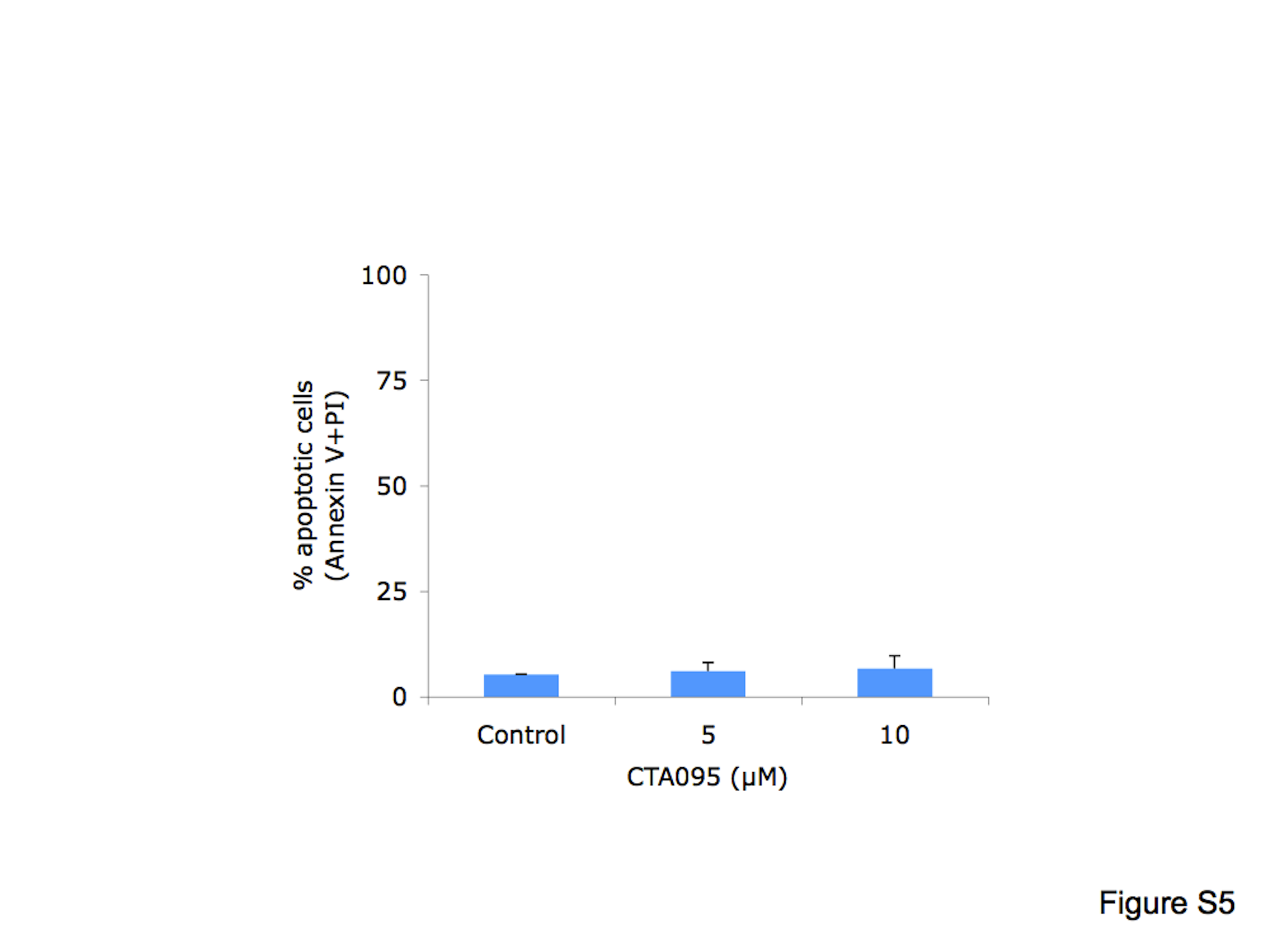

Supplement: Figure S5 — Induction of apoptosis of 293 cells following treatment with CTA095. 293 cells were seeded at 106 cells/ml (2 ml) in a 6-well plate overnight and then treated with CTA095 at the indicated concentrations for 24 h. Apoptosis was analyzed using Annexin-V FITC apoptosis detection kit. Columns, mean; bars, standard deviation, n = 3. (TIF) [file pone.0070910.s005.tif]
